# Sekundärer Wundverschluss mit einem neuen transparenten Unterdruckverband

**DOI:** 10.1007/s00104-023-01854-5

**Published:** 2023-05-12

**Authors:** Gunnar Loske

**Affiliations:** Alfredstr. 9, 22087 Hamburg, Deutschland

**Keywords:** Vakuumtherapie, Haut, Drainage, Folie, Wundmanagement, Vacuum therapy, Skin, Drainage, Film, Wound care

## Abstract

Sekundär heilende operative Wunden lassen sich mit der Unterdrucktherapie behandeln. Aufgrund der teilweise starken Adhärenz des in die Wunde eingelegten Polyurethanschaumes können Verbandwechsel schmerzhaft sein. Nach der Konditionierung und Débridement des Wundgrundes kann der sekundäre Wundverschluss operativ mit einer chirurgischen Naht erfolgen. Die kutane Unterdrucktherapie wird auch präventiv nach primärer chirurgischer Naht angewendet. Beschreibungen für sekundäre Wundverschlüsse ohne eine chirurgische Naht sind bislang nicht bekannt. Die Fertigung und Handhabung eines innovativen transparenten Verbandes für die kutane Anwendung der Unterdrucktherapie werden demonstriert. Die Verbandanordnung besteht aus einer transparenten Drainagefolie und einer transparenten Okklusionsfolie. Über eine Schlauchverbindung wird mit einer Unterdruckpumpe ein Unterdruck angelegt. Anhand eines Fallbeispiels wird eine neue Methode des sekundären Wundverschlusses unter Nutzung des transparenten Unterdruckverbandes vorgestellt. Der Behandlungszyklus mit einer Anleitung zur Herstellung des Verbandes wird im Video gezeigt.

**Video online:**

Die Online-Version dieses Beitrags (10.1007/s00104-023-01854-5) enthält ein Video. Im Video wird die Herstellung des transparenten Unterdruckverbandes gezeigt. Weiterhin wird der Behandlungszyklus des klinischen Fallbeispiels mit der Anwendung des TUV zum Wundverschluss einer sekundär heilenden Wunde demonstriert.

## Abstract (en)

Secondarily healing surgical wounds can be treated with negative pressure dressings. Dressing changes can be painful due to the strong adherence of the polyurethane foam placed in the wound. After debridement and conditioning of the wound bed, secondary surgical wound closure with a surgical suture can be performed. Cutaneous negative pressure treatment is used preventively after primary surgical suturing. Descriptions for secondary wound closure without a surgical suture are not known to date. The preparation and handling of an innovative transparent dressing for the cutaneous application of negative pressure treatment is demonstrated. The dressing assembly consists of a transparent drainage film and a transparent occlusion film. Negative pressure is applied via a tubing connection using a negative pressure pump. A new method of secondary wound closure using the transparent negative pressure dressing is presented based on a case example. The treatment cycle with instructions for making the dressing is shown in a video.

Sekundär heilende operative Wunden lassen sich mit der Unterdrucktherapie behandeln. Aufgrund der teilweise starken Adhärenz des in die Wunde eingelegten Polyurethanschaumes können Verbandwechsel schmerzhaft sein. Nach der Konditionierung und Débridement des Wundgrundes kann der sekundäre Wundverschluss operativ mit einer chirurgischen Naht erfolgen. Die kutane Unterdrucktherapie wird auch präventiv nach primärer chirurgischer Naht angewendet [1–3]. Beschreibungen für sekundäre Wundverschlüsse ohne eine chirurgische Naht sind bislang nicht bekannt.

Die Fertigung und Anwendung eines innovativen transparenten Verbandes für die kutane Anwendung der Unterdrucktherapie wird demonstriert. Anhand eines Fallbeispiels wird eine neue Methode des sekundären Wundverschlusses einer sekundär heilenden postoperativen Wunde unter Nutzung des transparenten Unterdruckverbandes (TUV) vorgestellt.

## Materialien und Methode

**Figure 1:**
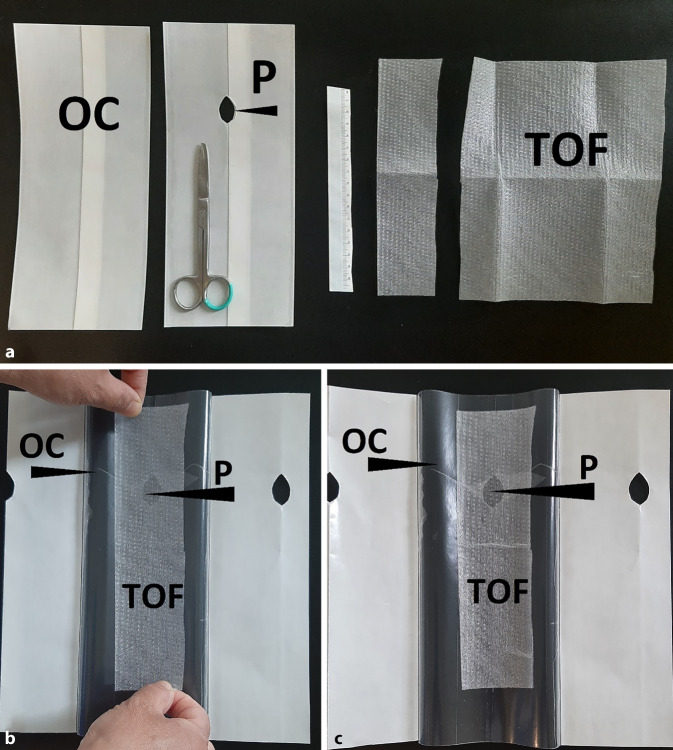

Die Basisform des TUV (Abb. 1) wird mit den folgenden Materialien hergestellt:

- transparente offenporige doppellagige Drainagefolie (TOF) (Suprasorb CNP Drainage Film, Lohmann & Rauscher International GmbH & Co. KG, Rengsdorf, Deutschland),
- transparente selbstklebende Okklusionsfolie (OC) (Suprasorb F, Lohmann & Rauscher International GmbH & Co.KG, Rengsdorf, Deutschland; V.A.C. Drape, KCI USA, Inc., San Antonio, Texas, USA),
- Schere.

Mit einer Schere wird die OC zugeschnitten. Die Maße richten sich nach der Größe der Wunde, die OC soll die Wundfläche zu allen Richtungen mehrere Zentimeter überlappen. In der Mitte wird eine ca. 2 cm durchmessende Öffnung (P) geschnitten. Hierüber wird später der Schlauchanschluss zur Unterdruckpumpe installiert.

Ein ca. 4–5 cm breiter Streifen der TOF wird in der passenden Länge der Wunde zugeschnitten.

Die Schutzfolie der selbstklebenden OC wird entfernt, sodass die Klebefläche freiliegt. Der TOF-Streifen wird so auf die Klebeseite geklebt, dass die P mit der TOF abdeckt ist. Mit diesem letzten Schritt ist die Verbandanordnung des TUV fertiggestellt und zum Einsatz bereit.

**Figure 2:**
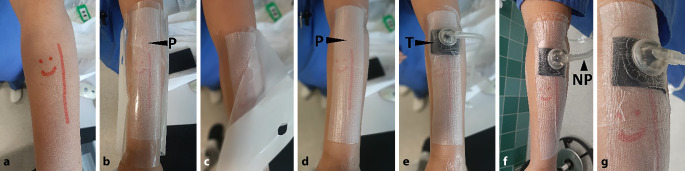

Der TUV wird in gleicher Technik wie ein üblicher Pflasterverband angelegt (Abb. 2). Die Verbandseite mit dem TOF-Streifen wird auf die Wunde aufgelegt und mit der selbstklebenden OC auf der Haut fixiert. Abschließend wird ein selbstklebender Schlauchanschluss (Trackpad) einer unterdruckerzeugenden Pumpeneinheit (ACTIV.A.C., KCI USA, Inc., San Antonio, Texas, USA; kontinuierlicher Sog −125 mm Hg) auf der Oberfläche des TUV über der P installiert. Über eine Schlauchleitung wird der Unterdruck über den TUV auf die Wunde geleitet. Der TOF-Streifen wird vollflächig auf die Wunde und die unmittelbare kutane Wundumgebung gesaugt (Kompression). Sekrete werden permanent aktiv abgeleitet (Drainage).

## Falldarstellung

**Figure 3:**
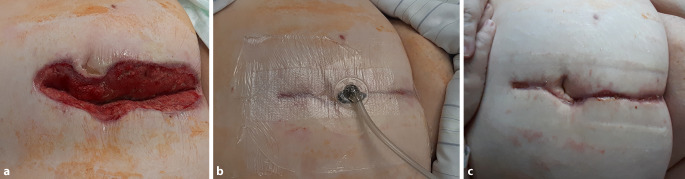

Anhand eines klinischen Fallbeispiels wird eine neue Methode des Wundverschlusses einer sekundär heilenden Wunde unter Nutzung eines TUV dargestellt (Abb. 3, Video).

Eine adipöse 62-jährige Patientin bedurfte aufgrund einer perforierten Sigmadivertikulitis der notfallmäßigen medianen Laparotomie mit Sigmaresektion. Im komplikativen postoperativen Verlauf kam es zu einer subkutanen Infektion der Laparotomiewunde. Die Wunde wurde komplett bis auf die geschlossene Fasziennaht geöffnet. Eine Unterdrucktherapie mit einem Polyurethanschaum und Folienokklusion wurde eingeleitet. Nach 10 Tagen und 2‑maligem Verbandwechsel zeigte die klaffende tiefe Wunde eine vollständig debridierte granulierende Wundoberfläche.

Die Unterdruckbehandlung wurde mit einem TUV weitergeführt. Durch eine manuelle Kompression von lateral wurden die Wundränder angenähert, sodass die klaffende Wundöffnung geschlossen war. Dann wurde der TUV aufgeklebt und die Schlauchverbindung zur Unterdruckpumpe mittels eines selbstklebenden Trackpads herstellt. Vor dem Aufkleben des Trackpads wurde noch ein kleiner Polyurethanschaumwürfel (V.A.C. Granufoam Dressing, KCI USA, Inc., San Antonio, Texas, USA; Suprasorb CNP Wundschaum, Lohmann & Rauscher International GmbH & Co. KG., Rengsdorf, Deutschland) als Sogvermittler auf die Folienöffnung aufgelegt. Mit einer Unterdruckpumpe (ACTIV.A.C; KCI USA, Inc., San Antonio, Texas, USA) wurde ein kontinuierlicher Unterdruck von −125 mm Hg angelegt.

Durch die Unterdruckausübung wurde die TOV vollständig flächig auf die Haut und Wunde gesaugt (Kompression). Gleichzeitig wurde das Wundsekret permanent abgesaugt (Drainage). Durch die Transparenz des TUV war jederzeit eine vollständige freie Sicht auf die Wunde unter der Therapie gegeben. Eine Entfernung des Verbandes zur Wundbeurteilung war nicht erforderlich.

Am 3. Tag erfolgte der erste Verbandwechsel. Der oberflächliche TUV ließ sich schmerzfrei entfernen. Die ehemals klaffende Wunde war bereits vollständig adaptiert und ohne klinische Zeichen einer Infektion. Auf der Wundfläche und der Haut, die im Saugkontakt mit dem TOF waren, sah man kleine regelmäßige Ansaugnoppen.

Die Unterdrucktherapie wurde für weitere 4 Tage fortgeführt. Hierzu wurde ein neuer TUV aufgeklebt und in der beschriebenen Technik an die Unterdruckerzeugung angeschlossen. Nach einer Gesamttherapiedauer von 7 Tagen wurde die kutane Unterdrucktherapie beendet. Der TUV wurde endgültig entfernt. Auf der Haut waren im Kontaktbereich mit dem TOF wieder die kleinen Ansaugnoppen zu sehen. Die Wunde war reizlos und über die gesamte Länge stabil adaptiert. Ein üblicher Feuchtverband wurde angelegt. In der Wundkontrolle am Folgetag waren die Saugveränderungen nicht mehr zusehen. Der vollständig dokumentierte Behandlungsverlauf wird im Video gezeigt.

## Diskussion

Der TUV ist ein neuartiger transparenter okklusiver Verband, an den ein Unterdruck angelegt werden kann.

Die Wundauflage der beschriebenen Verbandanordnung besteht aus einer doppellagigen offenporigen Drainagefolie (TOF). Die TOF ist ursprünglich für die intraabdominelle Unterdrucktherapie entwickelt worden [4, 5]. Die Drainagefolie besteht aus 2 transparenten Membranen, die mit unzähligen regelmäßig angeordneten und voneinander beabstandeten Perforationsöffnungen ausgestattet sind. Zwischen den beiden Membranblättern befindet sich ein Zwischenraum, der bei Sogausübung nicht kollabiert. Flüssigkeiten und Gase lassen sich durch die Poren und innerhalb des Zwischenraumes ableiten. Die Resorptionsfläche ist im Vergleich zum aufgewendeten Materialvolumen sehr groß. Unter Unterdruckausübung entfaltet sich der Unterdruck über die gesamte Folienoberfläche.

Das offenporige Drainagematerial der TOF kann anders als ein Polyurethanschaum intraabdominell im direkten Kontakt auf Peritonealorgane aufgelegt werden. Jüngst konnte unsere Gruppe zeigen, dass die TOF auch zur intrathorakalen Unterdrucktherapie beim Pleuraempyem zur Anwendung kommen kann [6].

Die Adhärenz der TOF auf Gewebe ist nicht so ausgeprägt wie bei Polyurethanschäumen. Aus der endoskopischen Anwendung ist bekannt, dass die Anhaftung einer Schaumdrainage auf einer granulierenden Wundoberfläche so stark sein kann, dass beim Entfernungsmanöver Schwamm- und sogar Abrisse der Drainageschläuche resultieren können [7, 8]. Seit einigen Jahren nutzen wir die TOF in der endoskopischen Unterdrucktherapie zur Herstellung von speziellen dünnlumigen Foliendrainagen, die das endoskopische Therapiespektrum erheblich erweitert haben [9–11]. Diese Foliendrainagen lassen sich immer leicht entfernen. Abrisse von Folienmaterial haben wir in zahlreichen Anwendungen noch nicht beobachtet.

Die TOF lässt sich als offenporiges Drainagematerial auch zur Unterdrucktherapie bei Wunden an der Körperoberfläche verwenden. Da der Verband weniger adhärent auf der Gewebeoberfläche ist, ist eine Entfernung beim Verbandwechsel fast schmerzfrei möglich. Die debridierenden Eigenschaften sind im Vergleich zu einem Polyurethanschaum geringer ausgeprägt. Auch diese Beobachtungen sind uns bereits aus zahlreichen Anwendungen in der endoskopischen Unterdrucktherapie bekannt.

Mit der gezeigten Verbandanordnung des TUV kann die TOF für alle Arten von Hautwunden, bei denen eine kutane Unterdrucktherapie angezeigt ist, genutzt werden. Der TUV kann auch zur Fortführung und Ergänzung der Unterdrucktherapie mit Polyurethanschäumen, in Kombination mit Polyurethanschäumen und gemeinsam mit der abdominellen Unterdrucktherapie eingesetzt werden. Eine Vielzahl von Anwendungsvarianten ist möglich. Die Handhabung und Fertigung des Verbandes sind einfach.

In der Unterdrucktherapie ist immer ein kreativer Umgang mit den vorhandenen Materialien erforderlich. Die Größe und Proportionen des Drainagematerials müssen angepasst werden. Aus der klinischen Anwendung hat sich der TUV entwickelt. Gemeinsam mit den Partnern der Industrie ist nach Lösungen zu suchen, um den Anwendern zukünftig standardisierte und zugelassene Medizinprodukte an die Hand zu geben, bei denen auch die formale Medizinproduktesicherheit vorliegt.

Am Fallbeispiel wird im Video eine Einsatzmöglichkeit des TUV für eine alternative Methode des Wundverschlusses von einer sekundären Wunde demonstriert. Zuvor war eine Unterdrucktherapie mit Polyurethanschäumen vorangegangen. Der herausragende Vorteil des TUV ist der Verzicht auf den operativen Verschluss mit einer Naht. Diese Innovation in der Nutzung der Unterdrucktherapie wird hiermit erstmals beschrieben.

Komplikationen wurden bei der kutanen Therapie nicht beobachtet. Denkbar ist, dass, wie auch beim operativen Wundverschluss, eine Infektion des verschlossenen Subkutangewebes auftreten kann. Dieses würde eine erneute Wunderöffnung und Erweiterung der lokalen Behandlung notwendig machen. Wie bei jedem offenporigen Drainagematerial können auch bei der TOF die Poren verstopfen und damit der TUV funktionslos werden. Auch eine Undichtigkeit des Verbandes führt zur Funktionslosigkeit, da eine Unterdruckausübung auf die Wunde nicht mehr besteht. Beide mögliche Störungen sind leicht durch eine visuelle Kontrolle des Verbandes bzw. durch einen Leckagealarm an der Unterdruckpumpe zu kontrollieren und durch einen Verbandwechsel zu beheben. Ein Nachteil könnte in der Fortführung der Unterdruckbehandlung mit der „Anbindung“ des Patienten an eine Verbandanordnung mit Drainageschlauch und Pumpe, die zu einer verlängerten Immobilität führen könnte, gesehen werden. Die praktische klinische Erfahrung aus zahlreichen Anwendungen seit 2017 zeigt hingegen, dass die Patienten durch einen TUV in ihrer Mobilität kaum eingeschränkt sind und die Akzeptanz des Verbandes sehr hoch ist. Denkbar ist die Verwendung von kleineren Pumpen, die Mobilität und den Patientenkomfort erhöhen. Ebenso ist es vorstellbar, dass die Behandlung nach der Erstanlage in einem ambulanten Setting fortgeführt werden kann. Hierdurch ließen sich stationäre Behandlungszeiten reduzieren und operative Kapazitäten, die sonst für den operativen Wundverschluss per Naht benötigt werden, freigeben.

Der herausragende Vorteil des TUV gegenüber allen anderen kutanen Unterdruckverbänden ist die transparente Eigenschaft des Verbandes. Jederzeit ist eine visuelle Kontrolle der Wundoberfläche und der Wundumgebung bei gleichzeitiger Unterdruckausübung möglich, ohne dass hierfür eine Entfernung des Verbandes notwendig wird.

## Fazit

Der transparente Unterdruckverband (TUV) ist ein neuartiger transparenter Wundverband, an den ein Unterdruck angelegt werden kann. Durch seine transparente Eigenschaft ist während der Behandlung eine vollständige visuelle Kontrolle der Wunde gegeben. Der Verband ist für kutane Wunden geeignet, bei denen eine Unterdrucktherapie zum Einsatz kommen kann. Mit der Verbandanordnung kann ein sekundärer Wundverschluss herbeigeführt werden.

## Supplementary Information





## Abkürzungen

NP: Unterdruck
OC: Transparente selbstklebende Okklusionsfolie
P: Öffnung
T: Trackpad
TOF: Transparente offenporige doppellagige Drainagefolie
TUV: Transparenter Unterdruckverband
